# Pegvisomant-Induced Cholestatic Hepatitis in an Acromegalic Patient with UGT1A1 ^⁎^ 28 Mutation

**DOI:** 10.1155/2016/2087102

**Published:** 2016-02-09

**Authors:** Maria Susana Mallea-Gil, Ignacio Bernabeu, Adriana Spiraquis, Alejandra Avangina, Lourdes Loidi, Carolina Ballarino

**Affiliations:** ^1^Servicio de Endocrinologıa, Hospital Militar Central, 726 Luis María Campos Avenue, 1425 Buenos Aires, Argentina; ^2^Endocrinology Division and Fundacion Publica Galega de Medicina Xenomica (Unidad de Medicina Molecular), Complejo Hospitalario Universitario de Santiago de Compostela, Universidad de Santiago de Compostela, Travesia Choupana s/n, Santiago de Compostela, 15706 La Coruña, Spain; ^3^Servicio de Gastroenterologıa, Hospital Militar Central, 726 Luis María Campos Avenue, 1425 Buenos Aires, Argentina; ^4^Departamento de Anatomıa Patologica, Hospital de Clínicas, Universidad de Buenos Aires, 2351 Córdoba Avenue, 1120 Buenos Aires, Argentina

## Abstract

Pegvisomant (PEGv) is a growth hormone receptor antagonist approved for the treatment of acromegaly; one of its documented adverse effects is reversible elevation of hepatic enzymes. We report a 39-year-old male acromegalic patient with a pituitary macroadenoma who underwent transsphenoidal surgery. The patient's condition improved but GH and IGF-I levels did not normalize; as a consequence, we first administered dopamine agonists and then somatostatin receptor ligands (SRLs) with poor response. PEGv 15 mg every other day was added to lanreotide 120 mg monthly. The patient developed a severe hepatitis five months after starting the combination therapy. Elevated ferritin, iron, and transferrin saturation suggested probable hepatitis due to haemochromatosis. We performed a liver biopsy which showed an acute cholestatic hepatitis consistent with toxic etiology. A heterozygous genotype* UGT1A1* ^⁎^ *28* polymorphism associated with Gilbert's syndrome was also found in this Argentine patient. The predominant clinical presentation resembled an acute cholestatic hepatitis associated with severe hemosiderosis, a different and new pattern of PEGv hepatotoxicity.

## 1. Introduction

Acromegaly is a rare disease usually derived from a GH-secreting pituitary tumor.

The increased mortality among persons with acromegaly includes higher prevalence of hypertension, hyperglycemia or overt diabetes, cardiomyopathy, and sleep apnea [1].

Currently, surgery is the primary approach for treating most patients. If cure is not achieved with this treatment, pharmacotherapy should be considered. Somatostatin receptor ligands (SRLs) are usually the first line therapy for treating active acromegaly. Pegvisomant (PEGv), a pegylated GH receptor antagonist, is another effective therapeutic option that could be administered alone or in combination with SRLs [2–4]. PEGv is usually well-tolerated; however, it might cause adverse events such as drug-induced liver injury [3].

Bernabeu et al. found that the* UGT1A1* ^⁎^ *28* genotype associated with Gilbert's syndrome predicts an increased incidence of liver abnormalities during PEGv therapy in Spanish acromegalic patients [4].

We report here clinical, biochemical, genotype, and histological findings in an Argentine acromegalic patient who developed a severe cholestatic hepatitis during PEGv therapy.

## 2. Case Report

In 1996 a 39-year-old man was referred to the Endocrinology Service of the Hospital Militar Central of Buenos Aires City because he presented arterial hypertension and acromegalic features. He had a two-year history of headaches, sweating, feet and hands enlargement, weight gain, erectile dysfunction, and hyperglycemia. His lab tests showed an elevated IGF-I level: >5.4 U/mL (NR: 0.6–5.4) and nonsuppressible GH postoral glucose tolerance test (OGTT) (GH nadir: 78 ng/mL). He underwent a magnetic resonance imaging (MRI) which showed a pituitary macroadenoma of 18 × 15 mm with suprasellar extension. The patient was operated on by transsphenoidal approach. The pathology report informed a diffuse eosinophilic pituitary adenoma. At that time we could not perform immunochemistry.

The symptoms and hormonal test improved; however, GH and IGF-I levels were persistently elevated.

In December 1996 bromocriptine 3.75 mg/day was indicated without normalization of IGF-I. Two years later, he was medicated with cabergoline 0.5 mg/week with poor tolerance and response; therefore, he returned to bromocriptine 5 mg/day. He stopped treatment and was lost to follow-up for five years. When the patient returned in 2003, an MRI showed no tumor but his IGF-I level was elevated; therefore, cabergoline was indicated. He was not consistent with the treatment because he presented poor tolerance to this drug. In 2005 he was started on octreotide 30 mg/month with partial response.

Due to severe coronary heart disease, in October 2007 the patient underwent a coronary bypass surgery. In December 2008, as he presented glucose intolerance, a more sensible diet and metformin (1,700 mg/day) were indicated.

In 2010 a cholecystectomy was performed because of the development of gallstones. In 2011 two benign polyps and a villous adenoma were removed during a colonoscopy.

**Figure 1:**
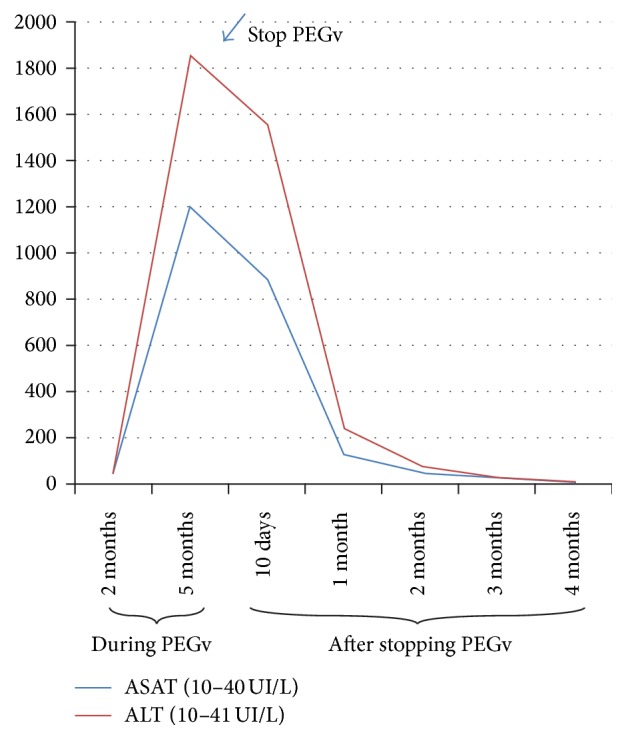
Chronological development of ASAT and ALT transaminases during the combination therapy with PEGv and after stopping this treatment.

In 2012 octreotide was switched to lanreotide 120 mg/month. Because of increased IGF-I levels, in March 2013 PEGv 15 mg every other day was added. Up to that moment the patient's liver function tests had always been normal. It is important to note that he did not drink alcohol. He was also medicated with atenolol 25 mg/day and enalapril 10 mg/day and continued metformin treatment. In May 2013 he presented transient symptoms of flu and a slight increase of transaminases, less than 3 times above the upper limit of normal (×ULN). In August 2013 the patient, under treatment with lanreotide and PEGv, had a normal IGF-I level, 154 ng/mL (81–225), but he unexpectedly experienced abdominal pain, severe asthenia, decreased appetite, and presented choluria the day before the visit to the hospital. Liver tests results showed elevated levels of aspartate aminotransferase (ASAT) 29.9 ×ULN, alanine aminotransferase (ALT) 45.3 ×ULN, alkaline phosphatase (APh) 2.26 ×ULN, and total bilirubin 2.5 ×ULN. Prothrombin time (PT) and activated partial thromboplastin time (aPTT) were both normal (PT: 85% (70–120) and aPTT: 39 seconds (27–42)). Blood cell count was normal: hematocrit 45%, hemoglobin: 15 grams/dL, and white blood cell count: 7100 cells/mcL with no eosinophilia. There were no changes in the normal pattern of coagulation tests and blood cell count during the course of the hepatic disease. The tests for viral and autoimmune hepatitis were negative. Elevated ferritin, iron, and transferring saturation (TSAT) suggested probable hepatitis due to haemochromatosis. Liver and iron tests are shown in Table 1 and in Figure 1. The abdominal ultrasonography was normal. We decided to stop treatment with both lanreotide and PEGv.

**Figure 2:**
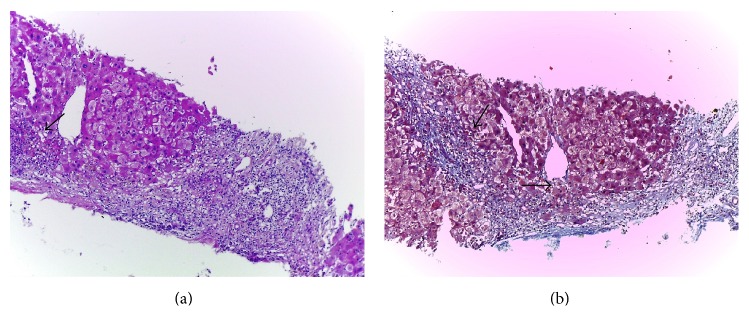
(a and b) Bridging periportal collapse with a mild number of inflammatory cells, HE, and Masson's trichrome, 100x, respectively. See arrows.

**Figure 3:**
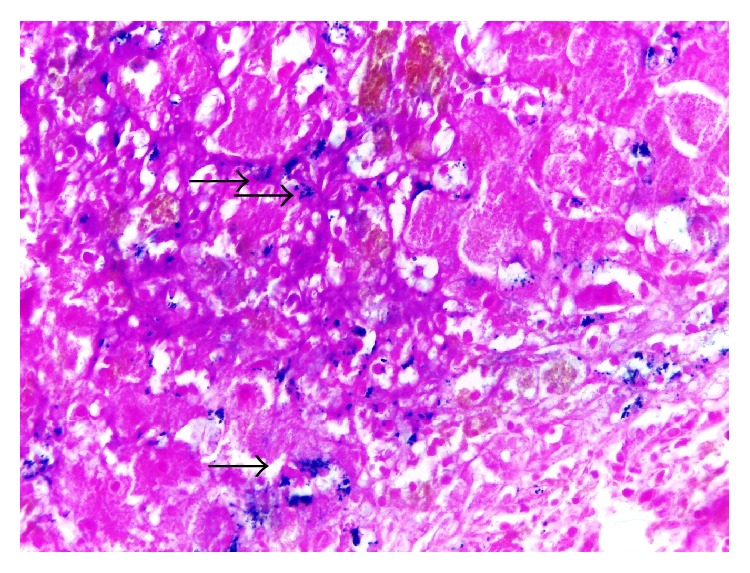
Moderate hemosiderosis: iron stores in reticuloendothelial cells, Perls stain, 400x. See arrows.

**Figure 4:**
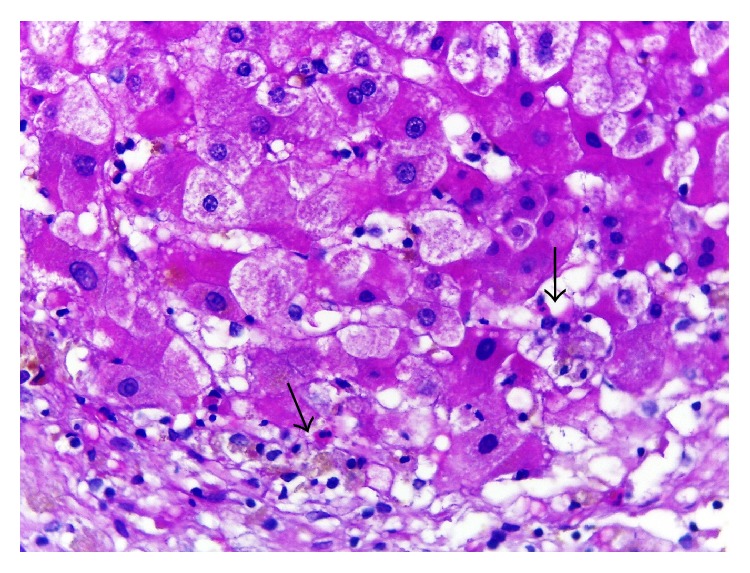
Focal acinar collapse. Sinusoidal inflammatory foci with some eosinophils. Hepatocyte cytoplasmic cholestasis, HE, 400x. See arrows.

We performed a liver biopsy which showed an acute cholestatic hepatitis with confluent necrosis, which formed portocentral bridges of parenchymal collapse (Figures 2(a) and 2(b)). There were deposits of iron predominantly in reticuloendothelial cells, a pattern which does not correspond to genetic haemochromatosis (Figure 3). Inflammatory cells included eosinophils (Figure 4). These findings were consistent with a severe acute hepatitis of toxic etiology.

Haemochromatosis was also ruled out through a negative result of C282Y and H63D genotypes of haemochromatosis HFE.

The genotype* UGT1A1* ^⁎^ *28* associated with Gilbert's syndrome was performed and a heterozygous genotype* UGT1A1* ^⁎^ *28* polymorphism was found. The* UGT1A1* ^⁎^ *28* genotyping was performed by PCR amplification of a fragment of the* UGT1A1* gene promoter, which included the TATA box, and Sanger sequencing of the amplification product.

Liver function tests returned to normal four months after PEGv discontinuation and they have been normal since then.

Without combination therapy, IGF-I levels increased to 367 ng/mL (NR: 81–225) (1.63 ×ULN). In January 2014 lanreotide 120 mg monthly and cabergoline 0.5 mg/week were indicated with good tolerance and thus IGF-I levels normalized: 205 ng/mL.

## 3. Discussion

The association of PEGv therapy with drug-induced hepatic events is well-documented; however, the mechanisms responsible for its toxicity remain unclear [3].

In the German Pegvisomant Observational Study, PEGv administration was associated with an increase in liver enzymes in 9% of patients [5]. ACROSTUDY reported that 30 (2.5%) of 1178 patients had elevated aspartate aminotransferase or alanine aminotransferase greater than three times the upper level of normal during PEGv therapy; a spontaneous recovery of transaminases frequently occurred despite continuation of PEGv [6].

According to Bernabeu et al., the* UGT1A1* ^⁎^ *28* genotype associated with Gilbert's syndrome predicts an increased incidence of liver abnormalities during PEGv therapy in Spanish patients. The excretion pathways of PEGv are unknown; in humans <0.6% of unchanged PEGv is excreted in urine. Nothing is known about the hepatic metabolism of PEGv and whether it requires UGT1A1-mediated glucuronidation [4, 5]. Although our patient had never shown increased bilirubin in order for us to be able to diagnose Gilbert's syndrome, we found a heterozygous genotype* UGT1A1* ^⁎^ *28* [7].

Drug-induced hepatotoxicity is a frequent cause of liver disease and can mimic all forms of acute and chronic hepatobiliary diseases. Based on ALT and APh levels, drug-induced liver injury is classified into acute hepatitis, cholestasis, or mixed patterns. This classification was established by the Council for International Organization of Medical Sciences and modified by US FDA Drug Hepatotoxicity Steering Committee [8]. Establishing its diagnosis requires exclusion of other causes of liver injury.

The liver is the central organ responsible for the selective uptake, metabolism, and excretion of drugs, xenobiotics, and environmental toxins. This essential function predisposes the liver to drug toxicity [9].

Adverse hepatic events caused by drugs can be considered to be either predictable (high incidence) or unpredictable (low incidence). The most common example of a drug causing predictable drug-induced liver injury (DILI) is acetaminophen. This type of drug injury has a short latency period, is dose-related, and is the most common form of DILI. On the contrary, idiosyncratic DILI is unpredictable, has longer/variable latency, and is less common [10].

The pathogenesis of drug-induced liver injury usually involves the participation of a toxic drug or a metabolite that either elicits an immune response or directly affects the biochemistry of the cell. Most adverse drug-induced hepatic events are unpredictable and are either immune-mediated hypersensitivity reactions or idiosyncratic ones [11].

The liver removes lipophilic chemicals from blood, including drugs, and biotransforms them into water-soluble metabolites which are excreted. This process involves cytochrome P450 (phase 1), conjugation (phase 2), and transport (phase 3). The expression of the enzymes and transporters involved in the hepatic handling of drugs are under the control of transcriptions factors (nuclear hormone receptors). In humans, polymorphisms of the genes that regulate each phase and transcription factors affect their activities and expression in response to environmental factors. So it is likely that the level of exposure to the toxic moiety (reactive metabolite or the parent drug) is the most upstream determinant of DILI [12].

Acromegaly by itself is correlated with a high prevalence of gallbladder stones and the long-term treatment with SRLs increases the incidence of cholelithiasis [13]. Our patient underwent a cholecystectomy, probably related to octreotide treatment. Five years later, at the onset of symptoms of hepatitis, his abdominal ultrasound was normal; that is why liver or biliary diseases were excluded.

Laboratory tests showed elevated ferritin and transferrin saturation suggesting probable hepatitis due to haemochromatosis, which was ruled out through a negative of C282Y and H63D genotypes and the liver biopsy. It is known that both ferritin and TSAT can also be elevated in many inflammatory conditions such as viral hepatitis, regular alcohol consumption, and fatty liver disease [14].

Filopanti et al. showed that in acromegalic patients treated with PEGv, multitherapies and previous episodes of liver disease were associated with increased risk of hepatotoxicity; however, they did not find any increased risk of liver toxicity in Italian patients with* UGT1A1* ^⁎^ *28* polymorphism [15]. Our patient was not medicated with any other probable hepatotoxic drug.

Neggers et al. found only 13.5% of transiently elevated liver transaminases (TET) above 3 ×ULN, of which 83% occurred within the first year of combination treatment with somatostatin analogs and PEGv. In their study, they detected the* UGT1A1* ^⁎^ *28* polymorphism in 54.2% of their patients; however, they could not confirm any association between this polymorphism and TET [16]. Our patient presented a slight increase of transaminases two months after starting the combination therapy and developed a severe hepatitis five months afterwards.

The presence of the* UGT1A1* ^⁎^ *28* genotype in our Argentine patient might be related to his Spanish ancestry (he had three Spanish grandparents). This finding is in accordance with Bernabeu et al. study in Spanish acromegalic population. Besides there is another coincidence with their study: our patient is male [4].

The mechanism of pegvisomant-induced liver injury remains the subject of conjecture. We report a patient with acromegaly who developed a severe drug-induced hepatitis during treatment with PEGv. When PEGv was discontinued, his laboratory tests normalized in four months.

Although in this case we might assume that it was an idiosyncratic drug toxicity, to the best of our knowledge, it is the first case described in which its predominant clinical presentation resembles an acute cholestatic hepatitis associated with severe hemosiderosis, a different pattern of PEGv hepatotoxicity.

## Figures and Tables

**Table 1:** Chronological development and values of alkaline phosphatase (APh), *γ*-glutamyltranspeptidase (GGT), total bilirubin (T. Bil), ferritin, transferrin saturation (TSAT), and iron during the combination therapy with PEGv and after stopping this treatment.

	During combination therapy		After stopping combination therapy				
	2 Months	5 Months	10 Days	1 Month	2 Months	3 Months	4 Months
APh (40–120 UI/L)	136	272	242	99	99	92	80
GGT (8–61 UI/L)			549	166	166	77	17
T. Bil (0.1–1 mg/dL)	0.4	2.5	1.9	0.64	0.64	0.61	0.61
Ferritin (30–400 ng/mL)			4836	763	595	374	191
Transferrin (200–360 ug/dL)			212	198	194	210	244
TSAT (15–50%)			104	72			50
Iron (59–158 ug/dL)			180	180			154
